# Incidence of venous thrombosis after peg-asparaginase in adolescent and young adults with acute lymphoblastic leukemia

**DOI:** 10.2217/ijh-2020-0009

**Published:** 2020-09-04

**Authors:** Brynne Underwood, Qiuhong Zhao, Alison R Walker, Alice S Mims, Sumithira Vasu, Meixiao Long, Tamanna Z. Haque, Bradley W Blaser, Nicole R Grieselhuber, Sarah A Wall, Gregory K Behbehani, James S Blachly, Karilyn Larkin, John C Byrd, Ramiro Garzon, Tzu-Fei Wang, Bhavana Bhatnagar

**Affiliations:** 1 Department of Internal Medicine, The Ohio State University, Columbus, OH 43210, USA; 2 Division of Hematology, Department of Internal Medicine, The Ohio State University, Columbus, OH 43210, USA; 3 The Ohio State University Comprehensive Cancer Center, Columbus OH 43210, USA

**Keywords:** acute lymphoblastic leukemia, adolescent young adult, peg-asparaginase, risk factors, venous thromboembolism

## Abstract

**Aim:**

There are limited data describing incidence of symptomatic venous thromboembolism (VTE) in adolescent and young adult (AYA) acute lymphoblastic leukemia (ALL) patients receiving peg-asparaginase.

**Materials & methods:**

Single-institution retrospective analysis of 44 AYA ALL patients treated with peg-asparaginase. Rates of VTE and proposed risk factors were assessed.

**Results:**

18 patients (41%) had a symptomatic VTE following peg-asparaginase. The cumulative incidence rate was 25% (95% CI: 13–38%) within 30 days of the initial dose. Personal history of thrombosis was statistically significantly associated with an increased risk of VTE with HR of 2.73 (95% CI: 1.40–5.33, p = 0.003) after adjusting for gender.

**Conclusion:**

These data indicate a high rate of VTE in the AYA ALL population following treatment with peg-asparaginase.

Asparaginase is an integral component of pediatric acute lymphoblastic leukemia (ALL) chemotherapy regimens and is known to be associated with improved long-term outcomes and survival [1,2]. Asparaginase aids in the hydrolysis of L-asparagine to L-aspartic acid and ammonia and glutamine to glutamic acid, which results in the depletion of L-asparagine and glutamine, both of which are necessary for maintaining rapid growth of lymphoblasts [3]. As such, the absence of these substrates subsequently leads to leukemia cell death and improved disease-free and overall survival for ALL patients [4].

Unfortunately, due to a number of serious drug-related toxicities, such as pancreatitis, hypersensitivity reactions, anaphylaxis and thrombosis, asparaginase is not universally included in adult ALL protocols [5]. Pediatric-inspired regimens for adolescent and young adult (AYA) patients with ALL, which contain either L-asparaginase or pegylated (peg)-asparaginase, have been demonstrated to improve disease responses and survival outcomes, and are now considered standard treatment for this age group, which includes patients 15–39 years of age per the National Comprehensive Cancer Network (PA, USA) AYA definition [6–9]. Fortunately, ALL remains a relatively rare disease in the AYA population, with a reported incidence of 1.1 per 100,000 cases [10].

One of the most significant asparaginase- related toxicities, which has high potential to affect morbidity and mortality, is venous thromboembolism (VTE). Although asparaginase has been noted to decrease procoagulant factors (such as fibrinogen, factors II, V, X), its predominant effect on coagulation is the reduction of natural anticoagulants, such as antithrombin (AT), as well as downregulation of fibrinolytic factors such as plasminogen thereby leading to a propensity for development of thromboembolic complications [11].

In pediatric ALL patients, symptomatic VTEs from asparaginase have been reported to be relatively low. A large meta-analysis by Caruso and colleagues reported a 5.2% rate of symptomatic thrombotic complications in 1752 pediatric ALL patients [12]. In their report, most VTEs occurred during induction chemotherapy and specific risk factors, such as lower asparaginase doses administered over longer periods of time, the use of prednisone over dexamethasone, and the presence of central venous catheters, were associated with a higher risk of thrombosis [12]. The PARKAA trial also reported a low rate of symptomatic deep vein thrombosis (5%) in pediatric ALL patients treated with L-asparaginase, however, asymptomatic deep vein thrombosis, which were detected prospectively using a number of screening imaging modalities following completion of L-asparaginase treatment, were diagnosed in 32% of patients [13].

In contrast, the incidence of asparaginase-related VTE in adult ALL patients have been shown to be consistently higher. Grace and colleagues demonstrated that the rate of asparaginase-related thrombosis increases with age in ALL patients treated across a number of Dana-Farber Cancer Institute (DFCI; MA, USA) protocols [14]. Whereas, only 5% of pediatric patients experienced symptomatic VTE, the rate of symptomatic VTE increased with age with rates of 20%, 25%, and 42% in the respective 11–14 years, 21–30 years and >30 years adult ALL patient age groups [14].

Although the existing literature on asparaginase-related VTE risks has mainly focused on pediatric or adult patient populations, VTE complications following peg-asparaginase administration in AYA ALL patients have not been extensively explored. However, in the general AYA oncology population, VTEs have been reported to occur in approximately 5% of patients across all cancer types [15]. The largest study to date to report outcomes following treatment with a pediatric-inspired protocol, CALGB 10403, reported a 5% rate of VTE in the 295 AYA patients who were enrolled. Additionally, half of these VTE events occurred within the context of an indwelling central venous catheter [9].

Given the relatively limited data regarding thrombosis complications from asparaginase within the AYA population and the observation that, at our institution, VTE appeared to be more common than expected, we conducted this retrospective study to further evaluate the incidence, risk factors, and outcomes of VTE in ALL patients in the AYA age group receiving peg-asparaginase.

## Materials & methods

### Patient characteristics

This is a single-institution retrospective study of AYA patients, between 18 and 38 years of age, with ALL who received peg-asparaginase as part of treatment per the CALGB 10403 [9] protocol, either on or off study, between January 2013 and December 2018. Most patients received the equivalent dose 2500 IU/m^2^ of peg-asparaginase per the CALGB 10403 treatment protocol. Off-study patients treated after 2014 received a peg-asparaginase dose that was capped at 3750 IU/dose due to increased toxicities above this dose. All VTE events in our patient population were diagnosed based on symptoms and confirmed with the appropriate imaging modality. VTE prophylaxis and screening were not considered standard of care in these patients at our institution. Clinical data, which included demographic characteristics, ALL diagnosis details, VTE risk factors, VTE characteristics, management of VTE, overall disease and survival outcomes, were collected by individual review of patient charts. The study was approved by the Institutional Review Board.

### Statistical analysis

Descriptive statistics such as medians and ranges, frequencies and percentages were used to summarize patient characteristics. Time to first VTE was calculated from the start date of ALL treatment to the date of first VTE onset, censoring at the transplant date or the last clinical visit date if no VTE event, treating death without VTE as a competing risk. For our population, the start date of ALL treatment represents when the first dose of peg-asparaginase was given, which was on day 4 of the treatment protocol. The cumulative incidence of VTE was estimated. The Fine and Gray regression models accounting for competing risks were used to examine the association between patient characteristics and risk of VTE. Overall survival (OS) was calculated from start of ALL treatment to death, censoring those alive at the last clinical visit date. Relapse-free survival (RFS) was time from start of ALL treatment to progression of ALL or death whichever occurred first, censoring those without progression or death at the last clinical visit date. Time to recurrence of VTE was calculated from the date of first VTE to the date of second VTE. The effect of VTE on OS and RFS were estimated by treating VTE as a time-dependent covariate in the regression model. Analyses were performed using Stata 14, and the statistical tests were two-sided with statistical significance defined as p <.05.

## Results

### Patient characteristics & risk factors for VTE

Patient characteristics and VTE risk factors are summarized in Table 1. 44 AYA patients with a diagnosis of B- or T-ALL or B- or T-lymphoblastic lymphoma (LBL) who received peg-asparaginase were included. There was one patient with lymphoid blast crisis due to progression from chronic myeloid leukemia included as well. The median age was 23.5 years (range, 18–38 years). 28 patients (64%) had B-ALL, 12 (27%) had T-ALL, three (7%) had T-LBL, and one (2%) had B-LBL. Only one patient (2%) had a personal history of VTE at the time of ALL diagnosis and three patients (7%) had a family history of VTE. 16 patients (36%) had a smoking history and, out of 16 female patients, two (13%) were on oral contraceptive pills at the time of ALL diagnosis. One patient was on norethindrone at the time of peg-asparaginase administration.

**Table 1.** Patient demographic and disease characteristics.

Characteristic	Total (n = 44)	VTE (n = 18)	No VTE (n = 26)	CALGB 10403 [9] (n = 295)
Sex: – Females – Males	16 (36.4) 28 (63.6)	3 (16.7) 15 (83.3)	13 (50) 13 (50)	115 (39) 180 (61)
Age: – Median – Range – 18–20 – 21–29 – 30–38	23.5 18–38 13 (29.6) 17 (38.6) 14 (31.8)	25 19–38 2 (11.1) 10 (55.6) 6 (33.3)	22.5 18–37 11 (42.3) 7 (26.9) 8 (30.8)	24 17–39 74 (25.1) 146 (49.5) 75 (25.4)
BMI kg/m^2^: – Median – Range – <30 – 30–40 – >40	24.5 18–48.2 36 (81.9) 2 (4.5) 6 (13.6)	24 18.8–40.2 16 (89) 1 (5.5) 1 (5.5)	25.4 18–48.2 20 (76.9) 1 (3.9) 5 (19.2)	26.5 14.9–54.3 201 (68.1) 72 (24.4) 22 (7.5)
Race: – C – AA – Other – Unknown	32 (72.7) 4 (9.1) 1 (2.3) 7 (15.9)	14 (77.8) 0 0 4 (22.2)	18 (69.2) 4 (15.4) 1 (3.9) 3 (11.5)	220 (74.6) 29 (9.8) 19 (6.4) 27 (9.2)
Personal history of thrombosis: – Y – N	1 (2.3) 43 (97.7)	1 (5.6) 17 (94.4)	0 26 (100)	N/A N/A
Family history of thrombosis: – Y – N	3 (6.8) 41 (93.2)	2 (11.1) 16 (88.9)	1 (3.9) 25 (96.1)	N/A N/A
Smoking history: – Y – N	16 (36.4) 28 (63.6)	7 (38.9) 11 (61.1)	9 (34.6) 17 (65.4)	N/A N / A
On OCPs: – Y – N	2 (4.5) 42 (95.5)	1 (5.6) 17 (94.4)	1 (3.9) 25 (26.1)	N/A N/A
Cell subtype: – B – ALL – T – ALL – B – lymphoblastic lymphoma – T – lymphoblastic lymphoma	28 (63.6) 12 (27.3) 1 (2.3) 3 (6.8)	13 (72.2) 4 (22.2) 0 1 (5.6)	15 (57.7) 8 (30.7) 1 (3.9) 2 (7.7)	223 (75.9) 71 (24.1) 0 0
WBC × 10^9^/l at diagnosis: – Median – Range – ≤30 – >30	8.8 0.4–520 31 (70.5) 13 (29.5)	5.3 0.71–82.6 16 (88.9) 2 (11.1)	17.4 0.4–520 15 (57.7) 11 (42.3)	9 0.4–444.6 217 (74.3) 75 (25.7)
Mediastinal mass at diagnosis: – Y – N	8 (18.2) 36 (81.8)	3 (16.7) 15 (83.3)	5 (19.2) 21 (80.8)	N/A N/A
Cytogenetics: – Unclassifiable – Intermediate – Unfavorable – Not fully evaluable – Favorable	17 (38.6) 18 (40.9) 5 (11.4) 4 (9.1) 0	7 (38.9) 9 (50) 1 (5.6) 1 (5.6) 0	10 (38.5) 9 (34.6) 4 (15.4) 3 (11.5) 0	25 (9.8) 112 (43.6) 18 (7) 86 (33.5) 16 (6.2)

N/A: Data were not evaluated in the CALGB 10403 population.

AA: African–American; ALL: Acute lymphoblastic leukemia; BMI: Body mass index; C: Caucasian; LBL: Lymphoblastic lymphoma; OCP: Oral contraceptive; WBC: White blood cell.

Patient characteristics and risk factors were comparable between our cohort and those treated on CALGB 10403 [9]. Notable differences included a lower percentage of patients with B-ALL in our cohort (64 vs 76%) as well as the absence of any patients with LBL in the CALGB 10403 cohort. We used the same cytogenetic risk assignment as CALGB 10403, which was based on criteria from the CALGB 19802 trial [16], to assign patients to favorable, intermediate, unfavorable, unclassifiable and not fully evaluable risk groups. The majority of our patients were in the unclassifiable (39%) or intermediate (41%) cytogenetic risk groups, while the minority were in the unfavorable (11%) or not fully evaluable (9%) risk groups. There were no patients with favorable-risk disease. Compared with the CALGB 10403 cohort, the majority of patients were in the intermediate (43.6%) and not fully evaluable (33.5%) cytogenetic risk groups, while the minority were in the unclassifiable (9.8%), unfavorable (7%) and favorable (6.2%) cytogenetic risk groups.

Univariable analysis to identify risk factors for VTE are presented in Table 2. Male sex and personal history of thrombosis were significantly associated with an increased risk of VTE, with a hazard ratio (HR) of 3.45 (95% CI: 1.01–11.86, p = 0.049), and HR of 3.50 (95% CI: 2.05–7.06, p <.01, respectively. In multivariable analysis model, personal history of thrombosis remained statistically significantly associated with an increased risk of VTE with HR of 2.73 (95% CI: 1.40–5.33, p = .003) after adjusting for gender.

**Table 2.** Univariable analysis of risk factors for venous thromboembolism.

Characteristics	HR	95% CI		p-value
Age at Dx	1.03	0.97	1.10	.30
Male vs female	3.45	1.01	11.86	.05
BMI at Dx	0.96	0.89	1.04	.33
WBC at Dx	0.99	0.97	1.00	.14
Mediastinal mass at Dx	0.82	0.25	2. 71	.74
ALL cell subtype, T vs B	0.65	0.24	1.76	.40
Personal H/o thrombosis	3.50	2.05	7.06	<.01
Family H/o thrombosis	2.75	0.61	12.34	.19
Smoking history	0.99	0.40	2.45	.99
On OCP	1.15	0.20	6.69	.88
Fibrinogen level at ALL Dx	1.00	1.00	1.01	.18
Platelet count at ALL Dx	1.00	1.00	1.01	.58

ALL: Acute lymphoblastic leukemia; BMI: Body mass index; Dx: Diagnosis; H/o: History of; HR: Hazard ratio; OCP: Oral contraceptive pills; VTE: Venous thromboembolism; WBC: White blood cell.

### Incidence & treatment of VTE

**Figure 1.**
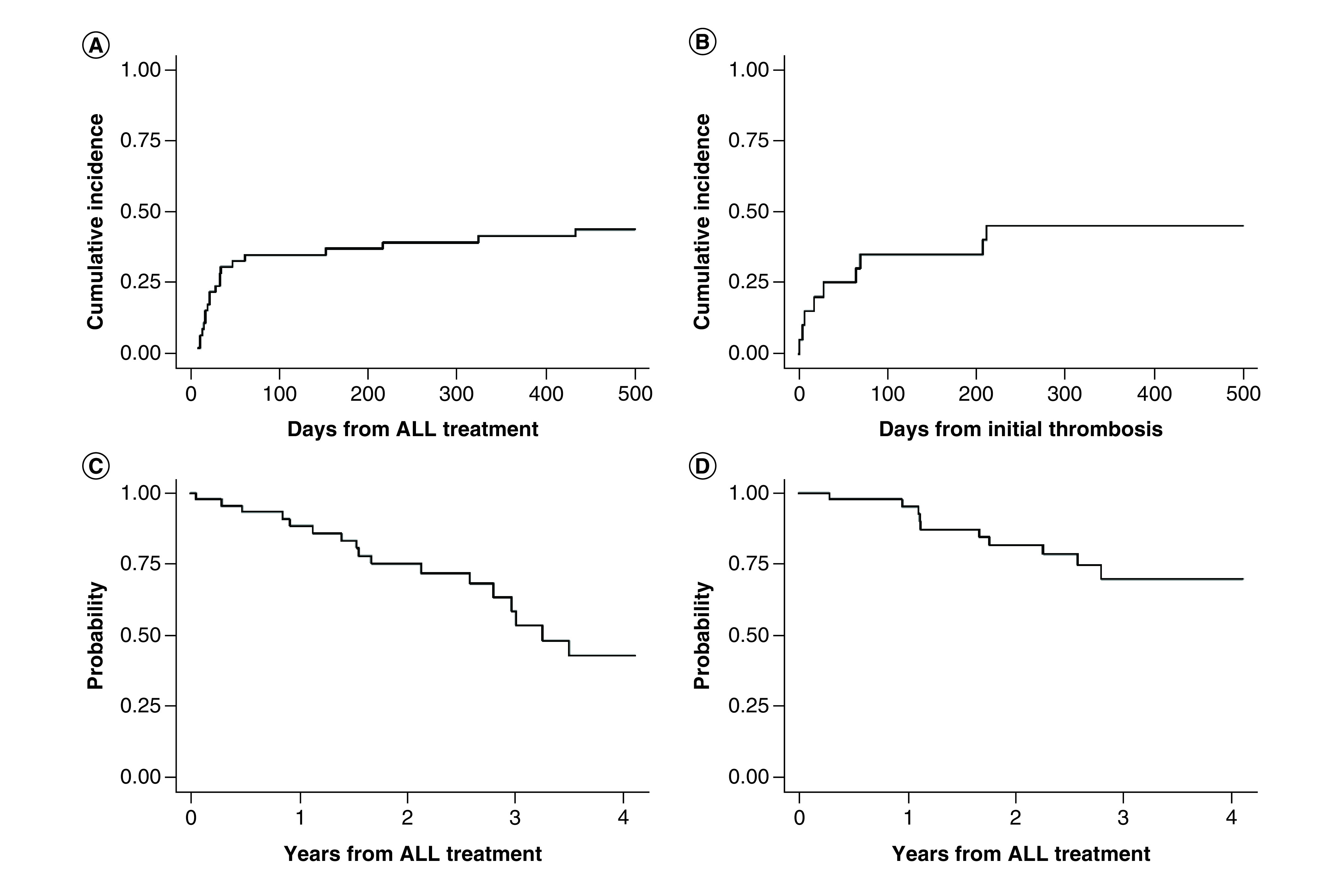
Venous thromboembolism incidence and disease outcomes of adolescent and young adult acute lymphoblastic leukemia patients receiving peg-asparaginase. **(A)** Cumulative incidence of VTE from start of ALL treatment, **(B)** cumulative incidence of recurrent VTE from initial VTE, **(C)** relapse-free survival from start of ALL treatment, **(D)** overall survival from start of ALL treatment. ALL: Acute lymphoblastic leukemia; VTE: Venous thromboembolism.

18 patients (41%) experienced a VTE complication following peg-asparaginase administration with a cumulative incidence rate of VTE of 25% (95% CI: 13–38%) within 30 days after their initial peg-asparaginase dose (Figure 1A). The initial VTE occurred most commonly during the induction phase of ALL treatment in thirteen patients (72%). Two patients (11%) experienced their first VTE during consolidation, two patients (11%) had a VTE during delayed intensification, and one patient (6%) had a VTE during maintenance. The median time to VTE onset was 14 days (range, 6–99 days) after the preceding dose of peg-asparaginase. On average patients received one dose of peg-asparaginase (range of 1–6) prior to diagnosis of initial VTE.

The VTE sites were upper extremity (n = 6), lower extremity (n = 6), cerebral (n = 6) and pulmonary (n = 1). One patient was diagnosed with two VTEs concurrently at differently sites and two (11%) patients had VTEs in association with a central line. The most common presenting symptom of initial VTE was edema and pain in ten out of the 18 patients (55.5%) who suffered from a VTE event followed by seizures in three (17%), focal findings on neurological exam in two (11%), shortness of breath in one (5.5%), headaches in one (5.5%) and altered mental status in one patient (5.5%). The most common diagnostic tests used to confirm the initial VTE event were upper and/or lower extremity dopplers in 11 (61%) out of the 18 patients who suffered from a VTE event followed by computed tomography (CT) pulmonary angiogram in one (6%), CT angiogram brain in two (11%), magnetic resonance imaging (MRI) brain angio-venogram in two (11%) and MRI brain with and without contrast in two (11%).

Antithrombin (AT) levels were obtained in ten patients at time of VTE diagnosis with a median activity level of 56.5% (35–94%). In patients who experienced at least one recurrent VTE, the median AT activity level was also 56.5% (35–72%). Fibrinogen levels (mg/dl) were obtained in 17 patients at time of VTE diagnosis with a median value of 130 (0–792) and a median value of 96 (0–792) in patients who experienced at least one recurrent VTE. Median platelet count (K/ul) at the time of VTE diagnosis was 124 (20–248) and a median count of 128 (20–226) in patients who experienced at least one VTE.

The initial VTE completely resolved in 13 patients (72%) with partial resolution in three (17%) and presence of a chronic thrombus in two patients (11%). Out of the 18 patients with VTE, nine (50%) had a total of 17 recurrent VTEs with the median (range) duration to recurrent VTE being 28 days (range, 1–211 days). The recurrent VTE events occurred most frequently during the consolidation phase (n = 7, 41%), followed by the maintenance phase (n = 6, 35%), induction (n = 3, 18%) and delayed intensification (n = 1, 6%). The most common location of recurrence was in a lower extremity vein (29% of total recurrent VTEs).

Only four patients (22%) who suffered from a VTE on peg-asparaginase received prophylactic anticoagulation, two received heparin and two received enoxaparin, prior to diagnosis of the initial VTE. In the absence of a standard institutional VTE prophylaxis guidelines, the most common reasons for patients not receiving VTE prophylaxis were presence or anticipation of prohibitory thrombocytopenia, increased bleeding risk or ambulating patient population. The most common initial treatment of VTE was a heparin infusion in nine patients (50%), followed by enoxaparin in seven patients (39%), and inferior vena cava filter placement in one patient (5.5%). The median total duration of anticoagulation therapy initiated after the initial VTE event was 203 days with the range being 21–560 days. Not included in this duration was one (5.5%) patient in whom anticoagulation was contra-indicated due to risk of bleeding.

The most common secondary thromboprophylaxis therapy for the seventeen patients (94%) who were initially placed on anticoagulation after their initial VTE was enoxaparin in 13 patients (76%). Other therapies included the initiation of enoxaparin along with inferior vena cava filter placement in two patients (12%) and direct oral anticoagulants in two patients (12%). Ten (56%) patients who experienced a VTE received at least one administration of AT. Nine (90%) of these patients received their first dose of AT after their initial VTE however one patient received AT as primary prophylaxis.

14 patients (32%) in the entire cohort experienced at least one bleeding complication, with nine of these patients suffering from at least one VTE. Five patients (36%) were on anticoagulation at the time of their bleeding complication. The most common initial bleeding complication involved the central nervous system in eight patients (57%), followed by ophthalmologic hemorrhages in five patients (36%) and hemorrhagic mucositis in one patient (7%). After initial VTE, peg-asparaginase was continued in ten patients (56%), stopped in six (33%), and dose reduced in two (11%).

### Overall survival & progression-free survival

At the time of data collection cut-off on 29 January 2020, 12 (27%) patients had died, with five deaths occurring as a result of ALL progression. The median follow-up duration among those alive was 36.1 months (range 3.4–71.8 months). Out of the seven patients with VTE for whom peg-asparaginase was discontinued or dose reduced, three (37.5%) developed progression of ALL compared with progression in two (18%) of the 11 patients in which peg-asparaginase was continued after VTE (p = .52). The 3-year RFS rate for the entire cohort was 62%. The RFS rates at years 1, 2 and 3 were 88.3% (95% CI: 74.1–95.0%), 75.3% (95% CI: 58.8–85.9%) and 61.1% (95% CI: 42.4–75.4%), respectively (Figure 1C). Median RFS duration was 42 months (95% CI: 33.5-not reached). The overall survival rates at years 1, 2 and 3 were 95.2% (95% CI: 82.2–98.8%), 82.2% (95% CI: 66.2–91.1%) and 72% (95% CI: 53.7–84.1%), respectively (Figure 1D). There was no significant difference in RFS or OS in those who did and did not experience VTE. In patients who experienced VTE, there was no significant difference in RFS or OS between those patients who got all of the planned doses of peg-asparaginase compared with those who did not get all planned doses.

## Discussion

This single-institution retrospective analysis shows an alarmingly high rate (41%) of VTE complications in 44 AYA patients treated with peg-asparaginase as part of a pediatric-inspired regimen for ALL. To our knowledge, this is the first analysis to focus specifically on VTE complications after peg-asparaginase administration in this population as the majority of other studies have included AYA patients within a broader age group of ALL patients [14,17,18]. Notably, the largest study to date to evaluate outcomes in AYA ALL patients receiving peg-asparaginase, CALGB 10403, reported only a 5% VTE rate, half of which were in association with the presence of an indwelling central venous access catheter [9]. In contrast, only 11% of our study population experienced VTE in association with a central line. Although our study and CALGB 10403 contained the same patient population, the vastly differing rates of VTE between the two studies is compelling and the reason for this difference is unclear. Our results are more aligned with those of the DFCI data in which the symptomatic VTE rate was observed to be 42% in ALL patients over 30 years of age; however, that study included a number of patients past the age of 39 years and outside the standard age range that is typically considered for AYA patients [14]. Furthermore, we observed a symptomatic VTE cumulative incidence rate of 42% (95% CI: 27–56%) by 216 days following the initial peg-asparaginase dose, which is significantly higher than the 18.1% cumulative incidence rate by 2.5 years that Rank and colleagues had observed in their AYA cohort [18].

We assessed a number of patient and disease related risk factors which could potentially explain the high number of VTEs in our AYA patients and were only able to identify personal history of thrombosis and male sex as risk factors for peg-asparaginase related VTE. Admittedly, there was only one patient in our study population who had a personal VTE history prior to therapy, which limits the impact of this particular finding but may lead to hypothesis-generating studies for future work. Male sex, however, has not been previously reported to predict increased risk of VTE in ALL patient populations. In large noncancer population studies, males have been reported to experience initial VTEs slightly more frequently than women (130/100, 000 vs 110/100,000, respectively) [19,20]. Additionally, Kyrle and colleagues showed that the risk of recurrent VTE was significantly higher in men than women (20 vs 6%) and that male sex was an independent risk factor for VTE recurrence [21]. It remains unclear whether hormonal fluctuations in this setting contribute to the differences in risk of VTE between sexes. Multiple other studies have identified different clinical risk factors (Table 3), and recent research further explored genetic risk factors of VTE in this population. For instance, the NOPHO ALL2008 group was able to create a genetic risk score based on the presence of single nucleotide polymorphisms, F11 rs2036914 and FGG rs2066865 that associated with higher risk of VTE particularly in adolescents (HR: 1.64) [22].

**Table 3.** Comparison of venous thromboembolism rates and risk factors in previously studied acute lymphoblastic leukemia patient populations who received peg-asparaginase.

Reference (study)	Population	n	VTE rate	Risk factors	Ref.
Caruso *et al.*, 2006 (meta-analysis)	Pediatric (<18 years)	1752	5.2% (sx)	• Induction phase • Lower doses of peg-asparaginase for longer periods and administration with anthracycline and prednisone • Central lines • Thrombophilic genetic abnormalities	[12]
Grace *et al.*, 2011 (DFCI)	Pediatric and adult (0–50 years)	548	8% All (sx) 5% Pediatric 34% Adult	• Age 30 years or older (VTE rate 42%)	[14]
Gugliotta *et al.*, 1992 (GIMEMA protocol ALL 0288)	AYA and adult (12–68 years)	238	4.2%	• Administration of peg-asparaginase with prednisone	[17]
Klaassen *et al.*, 2019 (DCOG ALL-10)	Pediatric (<18 years)	778	7.6%	• Age 7 years or older • T-ALL subtype	[23]
Lauw *et al.*, 2013 (HOVON-37)	Adult	240	10%	• VTE incidence was significantly lower with FFP supplementation than without FFP	[24]
Mitchell *et al.*, 2003 (PARKAA)	Pediatric (<18 years)	60	Asymptomatic 32% Symptomatic 5%	• Screened for VTEs at the end of asparaginase treatment • Symptomatic VTEs were confirmed by appropriate radiographic tests	[13]
Rank *et al.*, 2018 (NOPHOALL2008)	Pediatric and adult (1–45 years)	1772	7.9% All 3.7% 1–9.9 years 15.5% 10–17.9 years 18.1% adult	• Age 10 years or older • Enlarged lymph nodes • Mediastinal mass	[18]
Stock *et al.*, 2019 (CALGB10403)	AYA (17–39 years)	295	5%	• Central lines	[9]

ALL: Acute lymphoblastic leukemia; AYA: Adolescent and young adult; VTE: Venous thromboembolism.

Furthermore, it is possible that concomitant high dose steroid administration, specifically prednisone, during induction contributed to an increased risk of VTE. This association has been reported in both pediatric and adult ALL populations who have received peg-asparaginase [12,17,25]. However, other large randomized trials have shown no difference in VTE rates based on choice of corticosteroid [26,27]. In our cohort, out of the 18 patients who suffered from a VTE after peg-asparaginase, prednisone was given prior to the initial VTE in 16 (89%) patients and two (11%) patients received both prednisone and dexamethasone prior to the initial VTE. The relationship of prednisone to VTE may be related to its inhibition of fibrinolysis secondary to its ability to increase synthesis of plasminogen activator inhibitor and decrease levels of fibrinogen and plasminogen. Prednisone may have more of these effects compared with dexamethasone because of its mineralocorticoid properties which dexamethasone lacks [25,28]. Of important note, the successor trial to CALGB 10403, Alliance A041501 (NCT03150693), which is currently enrolling, replaces prednisone with dexamethasone during the induction phase which could, theoretically, reduce VTE rates.

Similar to Grace and colleagues [14], we observed no difference in OS and RFS between patients with and without VTE, however, we acknowledge that our small sample is too small to make any definitive statements regarding outcomes.

Expert guidelines have been proposed with respect to optimization of management for ALL patients receiving peg-asparaginase [29]. However, alternate doses and dosing schedules of peg-asparaginase have also been explored in an effort to reduce toxicities. Derman and colleagues were able to show, retrospectively, that patients who were treated per the CALGB 10403 regimen, off trial, with reduced doses of peg-asparaginase (≤1000 IU/m^2^) were able to achieve adequate asparagine depletion and fewer total grade 3 or 4 toxicities, compared with patients who received standard dose peg-asparaginase (doses ≥1000 IU/m^2^), without compromising RFS or OS [30]. However, rates of new VTE between the reduced dose peg-asparaginase group and the standard dose group were, essentially unchanged (20 vs 15.4%, p = 0.726). Larger prospective studies evaluating the utility of this approach would provide stronger rationale to adopt this strategy. Within a large pediatric population comprising over 600 patients, intermittent versus continuous peg-asparaginase dosing revealed significant reduction in all major peg-asparaginase-related toxicities within the intermittent dosing group without any difference in disease-free survival [31]. Similar approaches could potentially be explored in the AYA population.

In addition, the utility of thromboprophylaxis is also worthy of investigation. In our cohort, only four patients out of 18 who developed VTE in our cohort received thromboprophylaxis at the time of VTE, so evaluating the risks and benefits or thromboprophylaxis in this high risk population is essential. It's possible that our prophylactic strategy could be intensified, specifically with more consistent prophylaxis for patients with thrombocytopenia. Large randomized studies are ongoing to evaluate the effect of thromboprophylaxis with direct oral anticoagulants (NCT02369653) or low molecular weight heparin in pediatric ALL patients receiving peg-asparaginase [23]. Based on these findings, future decisions to incorporate thromboprophylaxis in AYA ALL regimens could be extrapolated. In addition, the use of AT concentrates to prevent VTE in peg-asparaginase-treated patients is also controversial. The recently published International Society of Thrombosis and Hemostasis SSC (NC, USA) guidance document suggests monitoring of AT level as well as repletion during peg-asparaginase therapy in the adult population after the literature showed a 60% risk reduction in VTE when implementing such strategies [32]. Whether the AYA population can benefit from a similar approach warrants further investigation.

Limitations of our study include a retrospective study design, relatively small patient population, lack of multivariate analysis and inconsistent management of patients receiving peg-asparaginase (e.g., varying doses of peg-asparaginase, decision to check AT levels prior to peg-asparaginase dose, decision to provide AT repletion). Additionally, there was marked provider variation in VTE management. In patients treated with anticoagulation, patient compliance with anticoagulation was also unknown, thereby affecting rates of recurrent VTE.

In summary, we report a high rate of VTE in our AYA ALL patients who have received peg-asparaginase as part of a pediatric-inspired multi-agent chemotherapy regimen. Future larger prospective studies focusing on risk factors and risk-adapted approaches in the management of the AYA population will allow for targeted prevention and treatment strategies for VTE as well as other peg-asparaginase-related complications.

**Summary points**

- In this series of 44 adolescent and young adult (AYA) patients with acute lymphoblastic leukemia (ALL) who received peg-asparaginase as part of treatment with a pediatric inspired chemotherapy regimen, symptomatic venous thromboembolism (VTE) occurred in 41% of patients, which is notably higher than what is reported in other studies.
- The cumulative incidence rate of VTE within 30 days following peg-asparaginase was 25% (95% CI: 13–38%), with a median time to VTE onset of 14 days (range, 6–99 days) after the preceding dose of peg-asparaginase.
- 50% of patients who were diagnosed with a VTE experienced recurrent VTEs.
- The only risk factors identified for increased risk of VTE were male sex with a hazard ratio (HR) of 3.45 (95% CI: 1.01–11.86, p = 0.049) and personal history of thrombosis (HR: 2.73 [95% CI: 1.40–5.3, p = 0.003]).
- Larger studies in the AYA ALL population are needed to identify risk factors which will allow targeted prevention and treatment strategies for VTE.

## References

[1] Amylon MD, Shuster J, Pullen J Intensive high-dose asparaginase consolidation improves survival for pediatric patients with T cell acute lymphoblastic leukemia and advanced stage lymphoblastic lymphoma: a Pediatric Oncology Group study. Leukemia 13(3), 335–342 (1999). 10086723 10.1038/sj.leu.2401310

[2] Pession A, Valsecchi MG, Masera G Long-term results of a randomized trial on extended use of high dose L-asparaginase for standard risk childhood acute lymphoblastic leukemia. J. Clin. Oncol. 23(28), 7161–7167 (2005). 16192600 10.1200/JCO.2005.11.411

[3] Earl M Incidence and management of asparaginase-associated adverse events in patients with acute lymphoblastic leukemia. Clin. Adv. Hematol. Oncol. 7(9), 600–606 (2009). 20020672

[4] Wetzler M, Sanford BL, Kurtzberg J Effective asparagine depletion with pegylated asparaginase results in improved outcomes in adult acute lymphoblastic leukemia: Cancer and Leukemia Group B Study 9511. Blood 109(10), 4164–4167 (2007). 17264295 10.1182/blood-2006-09-045351 PMC1885493

[5] Kantarjian H, Thomas D, O'brien S Long-term follow-up results of hyperfractionated cyclophosphamide, vincristine, doxorubicin, and dexamethasone (Hyper-CVAD), a dose-intensive regimen, in adult acute lymphocytic leukemia. Cancer 101(12), 2788–2801 (2004). 15481055 10.1002/cncr.20668

[6] Deangelo DJ, Stevenson KE, Dahlberg SE Long-term outcome of a pediatric-inspired regimen used for adults aged 18–50 years with newly diagnosed acute lymphoblastic leukemia. Leukemia 29(3), 526–534 (2015). 25079173 10.1038/leu.2014.229 PMC4360211

[7] Ram R, Wolach O, Vidal L, Gafter-Gvili A, Shpilberg O, Raanani P Adolescents and young adults with acute lymphoblastic leukemia have a better outcome when treated with pediatric-inspired regimens: systematic review and meta-analysis. Am. J. Hematol. 87(5), 472–478 (2012). 22388572 10.1002/ajh.23149

[8] Siegel SE, Advani A, Seibel N Treatment of young adults with Philadelphia-negative acute lymphoblastic leukemia and lymphoblastic lymphoma: hyper-CVAD vs. pediatric-inspired regimens. Am. J. Hematol. 93(10), 1254–1266 (2018). 30058716 10.1002/ajh.25229 PMC7521142

[9] Stock W, Luger SM, Advani AS A pediatric regimen for older adolescents and young adults with acute lymphoblastic leukemia: results of CALGB 10403. Blood 133(14), 1548–1559 (2019). 30658992 10.1182/blood-2018-10-881961 PMC6450431

[10] Society AC. Cancer Facts & Figures 2020 (2020). www.cancer.org

[11] De Stefano V, Za T, Ciminello A, Betti S, Rossi E Haemostatic alterations induced by treatment with asparaginases and clinical consequences. Thromb. Haemost. 113(2), 247–261 (2015). 25338526 10.1160/TH14-04-0372

[12] Caruso V, Iacoviello L, Di Castelnuovo A Thrombotic complications in childhood acute lymphoblastic leukemia: a meta-analysis of 17 prospective studies comprising 1752 pediatric patients. Blood 108(7), 2216–2222 (2006). 16804111 10.1182/blood-2006-04-015511

[13] Mitchell LG, Andrew M, Hanna K A prospective cohort study determining the prevalence of thrombotic events in children with acute lymphoblastic leukemia and a central venous line who are treated with L-asparaginase: results of the Prophylactic Antithrombin Replacement in Kids with Acute Lymphoblastic Leukemia Treated with Asparaginase (PARKAA) Study. Cancer 97(2), 508–516 (2003). 12518376 10.1002/cncr.11042

[14] Grace RF, Dahlberg SE, Neuberg D The frequency and management of asparaginase-related thrombosis in paediatric and adult patients with acute lymphoblastic leukaemia treated on Dana-Farber Cancer Institute consortium protocols. Br. J. Haematol. 152(4), 452–459 (2011). 21210774 10.1111/j.1365-2141.2010.08524.x PMC5763913

[15] O'brien SH, Klima J, Termuhlen AM, Kelleher KJ Venous thromboembolism and adolescent and young adult oncology inpatients in US children's hospitals, 2001 to 2008. J. Pediatr. 159(1), 133–137 (2011). 21353248 10.1016/j.jpeds.2011.01.005

[16] Stock W, Johnson JL, Stone RM Dose intensification of daunorubicin and cytarabine during treatment of adult acute lymphoblastic leukemia: results of Cancer and Leukemia Group B Study 19802. Cancer 119(1), 90–98 (2013). 22744771 10.1002/cncr.27617 PMC4220742

[17] Gugliotta L, Mazzucconi MG, Leone G Incidence of thrombotic complications in adult patients with acute lymphoblastic leukaemia receiving L-asparaginase during induction therapy: a retrospective study. The GIMEMA Group. Eur. J. Haematol. 49(2), 63–66 (1992). 1397242 10.1111/j.1600-0609.1992.tb00032.x

[18] Rank CU, Toft N, Tuckuviene R Thromboembolism in acute lymphoblastic leukemia: results of NOPHO ALL2008 protocol treatment in patients aged 1 to 45 years. Blood 131(22), 2475–2484 (2018). 29661787 10.1182/blood-2018-01-827949 PMC5981169

[19] Silverstein MD, Heit JA, Mohr DN, Petterson TM, O'Fallon WM, Melton LJ 3rd Trends in the incidence of deep vein thrombosis and pulmonary embolism: a 25-year population-based study. Arch. Intern. Med. 158(6), 585–593 (1998). 9521222 10.1001/archinte.158.6.585

[20] Tormene D, Ferri V, Carraro S, Simioni P Gender and the risk of venous thromboembolism. Semin. Thromb. Hemost. 37(3), 193–198 (2011). 21455853 10.1055/s-0031-1273083

[21] Kyrle PA, Minar E, Bialonczyk C, Hirschl M, Weltermann A, Eichinger S The risk of recurrent venous thromboembolism in men and women. N. Engl. J. Med. 350(25), 2558–2563 (2004). 15201412 10.1056/NEJMoa032959

[22] Jarvis KB, Leblanc M, Tulstrup M Candidate single nucleotide polymorphisms and thromboembolism in acute lymphoblastic leukemia – a NOPHO ALL2008 study. Thromb. Res. 184, 92–98 (2019). 31715544 10.1016/j.thromres.2019.11.002

[23] Klaassen ILM, Lauw MN, Van De Wetering MD TropicALL study: thromboprophylaxis in children treated for acute lymphoblastic leukemia with low-molecular-weight heparin: a multicenter randomized controlled trial. BMC Pediatr. 17(1), 122 (2017). 28486976 10.1186/s12887-017-0877-x PMC5424373

[24] Lauw MN, Van Der Holt B, Middeldorp S, Meijers JC, Cornelissen JJ, Biemond BJ Venous thromboembolism in adults treated for acute lymphoblastic leukaemia: Effect of fresh frozen plasma supplementation. Thromb Haemost 109(4), 633–642 (2013). 23364346 10.1160/TH12-11-0845

[25] Nowak-Gottl U, Ahlke E, Fleischhack G Thromboembolic events in children with acute lymphoblastic leukemia (BFM protocols): prednisone versus dexamethasone administration. Blood 101(7), 2529–2533 (2003). 12517808 10.1182/blood-2002-06-1901

[26] Möricke A, Zimmermann M, Valsecchi MG Dexamethasone vs prednisone in induction treatment of pediatric ALL: results of the randomized trial AIEOP-BFM ALL 2000. Blood 127(17), 2101–2112 (2016). 26888258 10.1182/blood-2015-09-670729

[27] Vrooman LM, Stevenson KE, Supko JG Postinduction dexamethasone and individualized dosing of *Escherichia Coli* L-asparaginase each improve outcome of children and adolescents with newly diagnosed acute lymphoblastic leukemia: results from a randomized study--Dana-Farber Cancer Institute ALL Consortium Protocol 00–01. J. Clin. Oncol. 31(9), 1202–1210 (2013). 23358966 10.1200/JCO.2012.43.2070 PMC3595424

[28] Ott N, Ramsay NK, Priest JR Sequelae of thrombotic or hemorrhagic complications following L-asparaginase therapy for childhood lymphoblastic leukemia. Am. J. Pediatric Hematol. Oncol. 10(3), 191–195 (1988). 10.1097/00043426-198823000-00002 3177809

[29] Stock W, Douer D, Deangelo DJ Prevention and management of asparaginase/pegasparaginase-associated toxicities in adults and older adolescents: recommendations of an expert panel. Leuk. Lymphoma 52(12), 2237–2253 (2011). 21827361 10.3109/10428194.2011.596963

[30] Derman BA, Streck M, Wynne J Efficacy and toxicity of reduced vs. standard dose pegylated asparaginase in adults with Philadelphia chromosome-negative acute lymphoblastic leukemia. Leuk. Lymphoma 61(3), 614–622 (2020). 31680584 10.1080/10428194.2019.1680839 PMC7028458

[31] Albertsen BK, Grell K, Abrahamsson J Intermittent versus continuous PEG-asparaginase to reduce asparaginase-associated toxicities: A NOPHO ALL2008 randomized study. J. Clin. Oncol. 37(19), 1638–1646 (2019). 30978155 10.1200/JCO.18.01877

[32] Zwicker JI, Wang TF, Deangelo DJ The prevention and management of asparaginase-related venous thromboembolism in adults: guidance from the SSC on Hemostasis and Malignancy of the ISTH. J. Thromb. Haemost. 18(2), 278–284 (2020). 31999063 10.1111/jth.14671

